# AI-Assisted Cotton Grading: Active and Semi-Supervised Learning to Reduce the Image-Labelling Burden

**DOI:** 10.3390/s23218671

**Published:** 2023-10-24

**Authors:** Oliver J. Fisher, Ahmed Rady, Aly A. A. El-Banna, Haitham H. Emaish, Nicholas J. Watson

**Affiliations:** 1 Food, Water, Waste Research Group, Faculty of Engineering, University of Nottingham, Nottingham NG7 2RD, UK; ahmed.rady1@nottingham.ac.uk (A.R.); n.j.watson@leeds.ac.uk (N.J.W.); 2 School of Chemistry and Chemical Engineering, University of Surrey, Guildford GU2 7XH, UK; 3 Teagasc Food Research Centre, Ashtown, D15 DY05 Dublin, Ireland; 4 Department of Plant Production, Faculty of Agriculture, Saba Basha, Alexandria University, Alexandria 5424041, Egypt; aly-elsawy@alexu.edu.eg; 5 Department of Soils and Agricultural Chemistry, Faculty of Agriculture, Saba Basha, Alexandria University, Alexandria 5424041, Egypt; drhaitham1976@alexu.edu.eg; 6 School of Food Science and Nutrition, University of Leeds, Leeds LS2 9JT, UK

**Keywords:** machine learning, digital manufacturing, cotton, colour vision system, quality assessment, semi-supervised learning, active learning

## Abstract

The assessment of food and industrial crops during harvesting is important to determine the quality and downstream processing requirements, which in turn affect their market value. While machine learning models have been developed for this purpose, their deployment is hindered by the high cost of labelling the crop images to provide data for model training. This study examines the capabilities of semi-supervised and active learning to minimise effort when labelling cotton lint samples while maintaining high classification accuracy. Random forest classification models were developed using supervised learning, semi-supervised learning, and active learning to determine Egyptian cotton grade. Compared to supervised learning (80.20–82.66%) and semi-supervised learning (81.39–85.26%), active learning models were able to achieve higher accuracy (82.85–85.33%) with up to 46.4% reduction in the volume of labelled data required. The primary obstacle when using machine learning for Egyptian cotton grading is the time required for labelling cotton lint samples. However, by applying active learning, this study successfully decreased the time needed from 422.5 to 177.5 min. The findings of this study demonstrate that active learning is a promising approach for developing accurate and efficient machine learning models for grading food and industrial crops.

## 1. Introduction

Automation of processes can increase efficiency, sustainability, and resilience. It also frees the workforce from mundane and repetitive tasks in order to focus on higher value and/or more creative tasks. Data and machine learning are increasingly being used to model and automate a wide range of processes including automated drug discovery [1], fault detection [2], process optimisation [3], fuel-cell lifetime management, and food safety inspection [4]. Assessing the quality of food and industrial crops post-harvest is an essential stage for determining market value, downstream processing needs, and the preservation of product shelf life and traceability. Traditional quality assessment methods are often destructive, leading to resource loss, time inefficiency, and impracticality for real-time applications [5]. In response, the application of machine learning models utilising images of harvested crops has emerged as a non-destructive and efficient approach, enhancing productivity, reducing labour demands, and mitigating human error. While machine learning models have been developed for this purpose [4,6], their practical deployment is hindered by the high cost of labelling the crop images to provide data for model training. This study evaluates methodologies aimed at alleviating the image labelling burden in the context of grading an industrial crop, specifically cotton. While the focus is on cotton, the findings of this study are applicable to address similar quality-assessment challenges encountered in the grading of other food and industrial crops.

Within the textile sector, the cotton industry is paramount, as it accounts for 90% of all natural fibres [7] and has an estimated global workforce of 350 million [8]. A key stage in the harvesting and processing of cotton lint is the evaluation of its economic value by assigning it a grade, which is determined by its processability (e.g., cleaning requirements) and quality (e.g., fibre colour and length and the presence of dried cotton leaves, seed coats, barks, grass, and dust) [9]. Evaluating Egyptian cotton is a complex process that is still implemented mainly through human intervention. Incorrectly grading the cotton lint results in over-processing, which can lead to cotton fibre breakage, reducing the value of the cotton [10]. Automating the evaluation of cotton would improve productivity and reduce labour requirements, while also reducing the chances of incorrectly grading cotton lint samples caused by eye fatigue and the influence of variable inspection conditions such as light [11,12].

High-volume instrument (HVI) systems are used to assess the process quality of ginned fibres (i.e., fibres separated from seeds) as well as the grade of the ginned fibres. The HVI is a fibre-testing device that uses various optical sensors (e.g., spectrophotometer, light scattering) and mechanical testing methods (e.g., tensile strength) to determine fibre length, strength, maturity, and micronaire, and has been employed to automate cotton grading since the 1970s [13]. However, the HVI has several drawbacks, most noticeable the high capital cost. In addition, it is a destructive sampling method, occupies a large floor area, is prone to temporal and spatial variations, and has a poor agreement with human classifiers for grading of cotton originating outside the USA, for example, Egyptian cotton [14,15]. Egyptian cotton grows only in the Nile River area and yields a highly resistant, long-staple cotton that absorbs colour well and makes fabrics produced from it brighter [16]. The Egyptian cotton industry faces various challenges (e.g., fraud and low productivity [17]). There is a need to create cost-effective automated solutions to evaluate Egyptian cotton. These solutions aim to enhance productivity, decrease fraudulent activities, and ultimately empower farmers who have been affected by decreasing export values.

Optical methods are emerging as predominate alternative systems for measuring cotton lint, either as replacements for or supplements to manual inspection and HVI measurement systems. Optical methods include the colorimeter [18,19], computer scanners [20,21], charge-coupled device (CCD) cameras [11,12,13,22,23,24,25,26], single-lens reflex cameras [27], thermal cameras [28], infrared spectrometry [29,30], microscopes [31], and optical spectrometry [15,20]. Cotton data extracted from image-measuring systems have been utilised to develop machine learning classification models to successfully classify US upland cotton [18,26], Chinese upland cotton [11], and Egyptian cotton [32] into different grades. Cotton lint grading standards are used to classify and assess the quality of cotton fibres based on various characteristics (e.g., fibre length, fibre strength, colour, and trash content).

A machine learning classification model is an example of supervised machine learning that fits (i.e., ‘trains’) algorithms to labelled datasets (i.e., ‘training data’) to sort data into different classes. Supervised learning is a machine learning paradigm for problems where the available training data consists of labelled examples, meaning each input data point (e.g., cotton lint sample image) has a known output (e.g., cotton grade). Supervised learning models can achieve high levels of model accuracy but are reliant on access to high-quality labelled training data, representative of all the variation in the sample domain, which can be time-consuming and expensive to collect.

A significant barrier to using machine learning to classify Egyptian cotton grade is ensuring data veracity [32], which requires a labour-intensive and costly image-labelling procedure. Labelling or grading of Egyptian cotton fibres requires authentication by the Cotton Arbitration and Testing General Organisation (CATGO) located in Alexandria Governate (Northwest Egypt) [33,34], which creates logistic difficulties for cotton traders in regions such as Upper Egypt that are located further away. Another challenge is the time cost for manual grading by Egyptian officials from CATGO, which takes on average 10–30 s per sample (unpublished data). Likewise, HVI systems also need an average of 30 s to perform a complete classification of a single cotton sample [35]. Previous examples of machine learning image cotton-classification models have required more than 3000 labelled images [11], which would take approximately 25 h to grade and label. Furthermore, the cotton lint grade values vary by year, due to harvest conditions, varieties, and other environmental and ginning factors (i.e., conditions such as machine maintenance or moisture content that affect the separation processes to separate cotton fibres from seeds and other impurities) [10]. Therefore, these classification models would require retraining each season using new labelled data to ensure the models are reflective of the current grading criteria. The time and cost associated with the yearly collection and labelling of cotton lint samples is a challenge when developing an automated Egyptian cotton lint grading solution. However, once trained, machine learning model predictions are almost instantaneous, representing significant time saving for classifiers if this solution was to be deployed.

Several machine learning methods have been tested to reduce the image-labelling burden associated with image classification of cotton. In a study by Li and Yang (2020), the use of ‘few-shot learning’ was explored to classify cotton pests, thereby assisting farmers in making quick decisions regarding the application of appropriate pesticides or other measures to protect crop yield and quality [36]. Few-shot learning is a machine learning approach that enables models to learn and make predictions with very limited labeled data, often consisting of just one or a few examples per class. However, few-shot models may struggle to handle complex tasks that require more nuanced or fine-grained distinctions between classes (e.g., the overlap in measurements between Egyptian cotton grade [32]), as they lack sufficient training data [37]. Alternatively, semi-supervised learning and active learning are two machine learning methods that have proven successful in reducing the volume of labelled training data required. Semi-supervised methods construct the predictive model by learning from a few labelled training examples and a large pool of unlabelled data points [38]. By leveraging the additional unlabelled data, the models can learn a more accurate representation of the underlying data distribution, which can improve the models’ ability to generalise to new, unseen data. Within the textile industry, semi-supervised methods have been used to segment textile images with limited human assistance [39] and for fault detection in fabrics [40]. However, it is important to note that semi-supervised learning may not always improve the accuracy of a model, especially if the unlabelled data are noisy or irrelevant to the task at hand [41]. An alternative method to address these challenges is active learning. Active learning is a machine learning technique that allows models to interactively query a human to obtain additional labelled data to improve their performance [42]. The model actively identifies which data points to request labels for, based on its current uncertainty or confidence in its predictions [42]. Active learning can be particularly effective in scenarios where labelled data are scarce or expensive to obtain, and where the model’s performance can be improved by obtaining additional relevant and informative labels. This is needed as the time cost of labelling additional cotton lint samples is high (10–30 s per sample). If the additional number of additional samples is too many, it may not be feasible to use active learning to improve the model’s performance.

The objective of this research article is to compare the efficacy of supervised learning, semi-supervised learning, and active learning for the autonomous grading of Egyptian cotton lint samples from colour images. Previous research has demonstrated the development of classification models for this purpose; however, their practical deployment is not feasible due to high labelling costs. This study extends the prior work by examining the capabilities of semi-supervised and active learning to minimise labelling effort while ensuring high classification accuracy. The conclusions drawn from this study work are transferable to solve quality-assessment challenges encountered when grading food and other industrial crops.

## 2. Materials and Methods

### 2.1. Cotton Lint Samples

Cotton lint samples were provided by the Cotton Arbitration and Testing General Organization (CATGO), Alexandria, Egypt. All of these samples were harvested in 2020. The CATGO is responsible for providing the official certificates for authenticating cotton lint in terms of determining the quality attributes and grade of Egyptian cotton cultivars for cotton ginning companies. To ensure data veracity, human experts from CATGO labelled the samples provided, using the Egyptian cotton grading system. To aid communication, the grades were assigned a value from *I* for the highest quality grade to *IX* for the lowest quality grade. A total of 2261 labelled samples were provided, but unfortunately, not all grades were represented in the samples provided. The breakdown of the number of samples of each grade for each cultivar is reported in Table 1.

**Figure 1:**
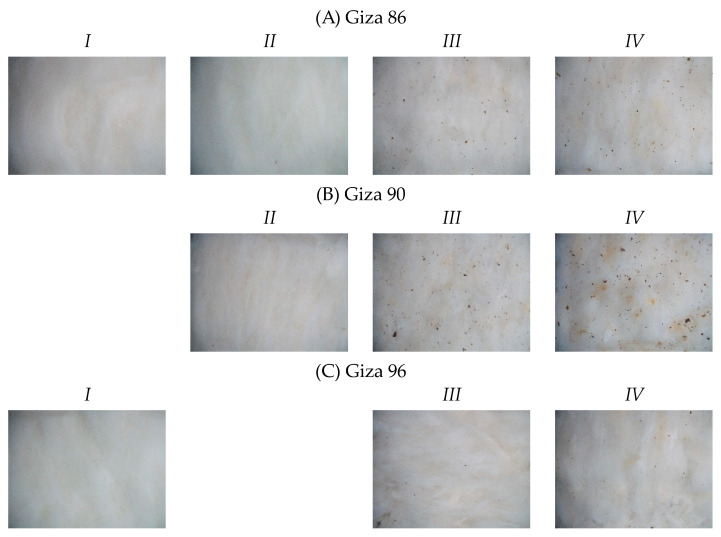
Sample images from Egyptian cotton grades I-IV that were available within the dataset for the cultivars (**A**) Giza 86, (**B**) Giza 90, and (**C**) Giza 96.

**Figure 2:**
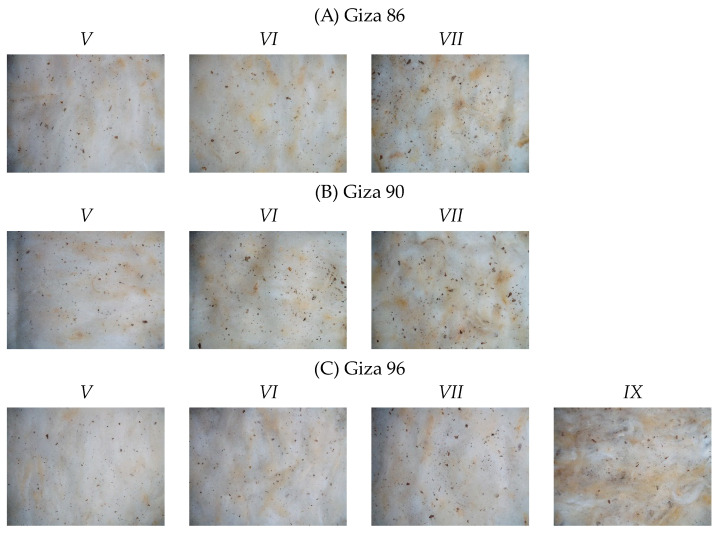
Sample images from Egyptian cotton grades V-IX that were available within the dataset for the cultivars (**A**) Giza 86, (**B**) Giza 90, and (**C**) Giza 96.

Samples from three cotton cultivars were collected, including cultivars from both staple categories of Egyptian cotton, long and extra-long. Giza 86 and 90 are long stable cultivars and Giza 96 is an extra-long staple cotton cultivar. Figure 1 and Figure 2 present sample images captured using the colour vision system outlined in Section 2.2.

### 2.2. Colour Vision System

Full details of the colour vision system can be found in Fisher et al. (2022) [32], and for brevity, only a summary is provided here. The colour vision system consisted of a CCD sensor 8.1 MP Fuji A850 digital camera (FUJIFILM Corporation, Minato-ku, Tokyo, Japan), mounted 11 cm vertically above the surface of the cotton sample. Consistent illumination of samples was ensured using a square 25.0 mm × 25.0 mm × 3.85 mm 10 W light-emitting diode (LED) light source (Intelligent Group Solutions Ltd., Thatcham, UK) also mounted 11 cm above the sample. To further ensure consistent illumination conditions, the camera and light source were enclosed in an aluminium box, whose dimensions were 54 cm × 40 cm × 40 cm and insides were coated black to minimise surface reflection. Samples of 4 cm thickness were placed directly below the sensor, and images were captured with no flash and stored in JPEG format with dimensions of 2248 pixels × 3264 pixels. Each image was transferred to a PC for analysis using Windows 10 operating software (21H2 10.0.19044.1645, Windows 10, Microsoft Corporation, Redmond, Washington, DA, USA).

### 2.3. Image-Processing Method

This study used the image-processing method developed in Fisher et al. (2022) [32] that evaluated multiple feature-extraction methods from cotton lint images. The best method determined in that work was to extract features describing the colour of the cotton lint and the percentage of trash present within the samples, as well as the intra-sample variation [32]. First, to eliminate the areas of the sample without uniform light, the samples were cropped from 2448 pixels × 3264 pixels to 2448 pixels × 2965 pixels. The number of pixels with presence of trash was detected by converting the cropped image to a grayscale image and then to a binary image using the balanced histogram thresholding method. The percentage of trash detected was then determined by dividing this value by the total number of image pixels. The colour of the cotton was determined by converting the cropped image from the original red–green–blue (RGB) colour space to the International Commission on Illumination (CIE) XYZ colour space and then the three-dimensional (3D) Hunter colour space using the equations outlined in Heng et al. (2020) [24]. The three extracted 3D Hunter parameters that were used to characterise the cotton lint colour were lightness (L*), relative similarity to the green–red (a*), and relative similarity to the blue–yellow (b*). The intra-sample variation of the sample was determined by segmenting the cropped image and then measuring the colour and detected trash values of each segmented image. The mean and standard deviation of these values were then calculated. All the image processing methods were conducted using MATLAB software (R2022a version, Mathworks, Natick, MA, USA).

### 2.4. Machine Learning Methods

Three machine learning methods, (1) supervised learning, (2) semi-supervised learning, and (3) active learning, were employed to develop models that used the cotton lint image data (input data) to classify the Egyptian cotton grade (output data). The goal of using semi-supervised learning and active learning methods was to reduce the volume of labelled data required to develop the models, while still achieving a comparable accuracy (within ±5% accuracy) to the supervised learning model.

Firstly, the data were partitioned into multiple training and testing datasets using stratified nested cross-validation. Cross-validation splits the data into ‘k’ number folds of training and testing datasets. Each of the folds is given an opportunity to be used as a held-back test set, whilst all other folds collectively are used as a training dataset. Stratified random sampling was used to ensure that the training and testing datasets were balanced across the Egyptian cotton grades [43]. Nested cross-validation is when an additional inner loop of cross-validation is used to optimise the model hyperparameters during model training. A hyperparameter is an adjustable algorithm parameter that must be either manually or automatically tuned in order to obtain a model with optimal performance. Using nested cross-validation, the risk of overfitting during hyperparameter optimisation was reduced, as the models were only exposed to a subset of the dataset provided by the outer cross-validation procedure [44]. The outer loop testing data were withheld until the end to evaluate the final models and then averaged to provide an unbiased evaluation of the models’ predictive capabilities. For the nested cross-validation, a k value of 10 was chosen for both the inner and outer folds because of the limited number of data points [45].

The models were trained using a random forest algorithm. Random forest models have been demonstrated more effective at predicting Egyptian cotton grade when compared with artificial neural networks and support vector machines, as they excel at multi-class classification problems [32]. The random forest is non-linear machine learning algorithm that uses multiple decision trees and a statistical technique called “bagging”, which randomly samples from the training data for each tree and randomly subsets the input variables at splitting nodes until the minimum node size is reached [46].

Semi-supervised learning and active learning require labelled and unlabelled datasets. Therefore, to recreate the conditions under which these learning methods are used, the training data were further partitioned into labelled and unlabelled data. To explore the effect of the volume of starting labelled data, sometimes referred to as “*seed data*”, on the learning methods, models developed using various ratios of labelled to unlabelled data ranging from 10:90 to 90:10 were evaluated. The labelled data were normalised to ensure all variables were given equal weight by the classification algorithms. To normalise without any loss of information, the minimax function was applied. The normalisation parameters were saved and then used to normalise the unlabelled data and testing data. Next, Bayesian optimisation was performed to find the optimal hyperparameters for the random forest [47]. For the Bayesian optimisation, the ‘expected improvement’ acquisition function was used to evaluate the expected amount of improvement in goodness of fit to the data and was stopped at 30 iterations. The random forest hyperparameters’ initial values were randomly set at the start of optimisation to explore a wider search space and increase the chances of finding better configurations efficiently. The hyperparameters optimised were: number of trees; maximum depth of each decision tree in the forest; minimum number of samples required to split an internal node; minimum number of samples required to be at a leaf node; maximum number of features to consider when looking for the best split; and maximum number of samples to draw from the dataset to train each base estimator. The model was then retrained using the best hyperparameters. When following the supervised learning method, each model developed in the inner loop was evaluated on the outer-loop testing data. The average of the classification accuracy across the folds was then calculated to provide the overall classification accuracy of the model.

Using the semi-supervised learning method, the model was then used to predict the labels of the unlabelled data set. The most confident prediction for each classification (e.g., Egyptian cotton grade) was then identified and, if the confidence in the prediction was above a user-specified limit, the prediction was used to label their respective unlabelled input data points. The random forest models’ prediction confidence was determined using the fraction of votes cast by the trees in the ensemble for each class label. The model was then retrained with the expanded labelled dataset, following the method of hyperparameter optimisation previously explained. This was repeated until either no more unlabelled data points remained or none of the predictions were above the user-specified confidence limit. Active learning followed a similar method; however, instead, the least confident predictions for each class were identified. If these confidence predictions were below a user-specified confidence limit, these predictions were manually labelled and added to the labelled data and the model was retrained using the expanded dataset. Again, this process was repeated until either no more unlabelled datapoints remained or none of the predictions were below the user-specified confidence limit. To investigate what effect confidence limit had on the models’ classification accuracy and training time, five different confidence limits were explored: 10%, 25%, 50%, 75%, and 90%. A flowchart summarising the three machine learning methods is provided in Figure S1. All the models were developed using MATLAB software (R2022a version, Mathworks, Natick, MA, USA).

## 3. Results and Discussion

### 3.1. Supervised Learning Results

**Figure 3:**
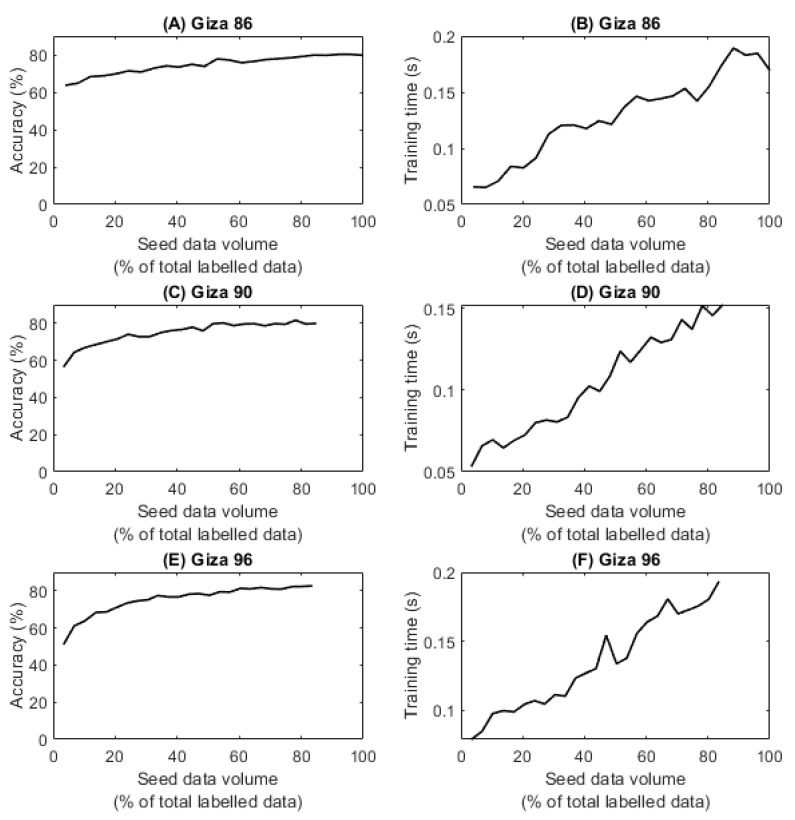
Supervised learning model prediction accuracy and training time for different volumes of training images (“seed data volume”) and cotton cultivars.

**Figure 4:**
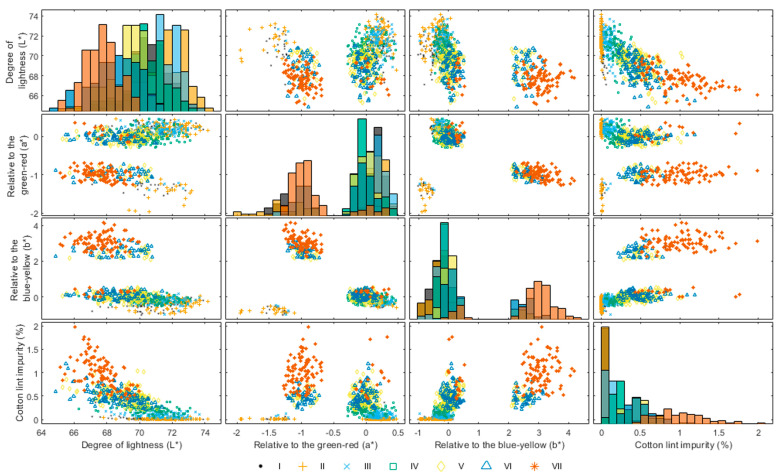
Scatter plot matrix showing the three-dimensional Hunter colour values lightness, L*, relative similarity to the green–red, a*, and relative similarity to the blue–yellow, b*, plotted against the percentage of trash detected. Data extracted from the Giza 86 cotton lint images for grades I to VII.

Benchmark supervised machine learning models were first developed to act as a standard against which to compare the semi-supervised and active learning models. The nested cross-validation classification accuracy and training-time results of the developed random forest models using different volumes of labelled “seed data” are presented in Figure 3. The maximum observed model accuracy for the cultivars Giza 86, 90, and 96 was 80.20%, 81.62%, and 82.66%, respectively. These results are slightly lower (~2%) than previously reported Egyptian cotton classification models developed using the same data [32]. This is likely to be explained due by this study training the models using nested cross-validation rather than a simple training and testing data split. Nested cross-validation is a more thorough training procedure, which means that the models may be more conservative in their predictions, as they are being evaluated on multiple different partitions of the data. In contrast, a simple training and testing data split may allow the model to overfit to the training data, leading to higher accuracy on the testing data but lower accuracy on new, unseen data [48]. Notwithstanding, the attained model accuracies (ranging from 80.20% to 82.66%) exhibit comparability, albeit towards the lower end, to accuracy results previously reported (ranging from 88.0% to 94.0%) using image processing and machine learning methods for the classification of US cotton lint [18,26]. The scatter-plot matrix is presented in Figure 4, which displays the pairwise scatter plots of multiple variables in the Giza 86 data. Figure 4 demonstrates that the decreased accuracy might result from the insufficiently distinct clustering of Egyptian cotton grades, where points in close proximity within the input space could pertain to distinct grades. This may be due to the colour vision system and image-processing methods not capturing the fibre lengths within the images, which is an important characteristic when grading Egyptian cotton lint samples [16,49]. Additionally, it could be the result of human error in the labelling of the dataset, which can be caused by fatigue, a common problem with repetitive labelling of image samples [11,12]. This observation is repeated for the Giza 90 and 96 plots within the Supplementary Information (SI). As reported in previous work [32], the cultivar fibre length, long or extra-long, appears not to have affected model accuracy, probably due to the colour vision system and image-processing methods not capturing the fibre lengths within the images.

A supervised learning model’s accuracy typically increases as more training data are provided, because the model has more examples to learn from and can better capture the patterns and relationships in the data, leading to better generalisation and prediction performance. This pattern is reflected in the results displayed in Figure 3. However, it can also be observed that the models’ accuracy appears to plateau as more labelled training data are provided. This may be due to a combination of factors, including:

1. Limited model capacity: In some cases, the model’s capacity may not be enough to handle the increased amount of data. When the model’s complexity is insufficient to capture the patterns in the data, adding more data may not improve the accuracy [50]. It may be that the random forest models developed within this study are not complex enough to process more data and future work should explore other models, such as deep learning that achieved an accuracy of 98.9% when classifying Chinese upland cotton grade [11].
2. Data quality: The quality of the new data added to the training set can affect the model’s accuracy. If the new data are noisy, inconsistent, or biased, they may not contribute much to the model’s accuracy and may even degrade it. Previous work highlighted the presence of human error within the labelling of Egyptian cotton data used to train the models [32], implying that data quality may be causing the models’ accuracy to plateau.
3. Data redundancy: As the amount of training data increases, some of the data may become redundant or provide little new information to the model. This can lead to a plateau in accuracy as the model is not gaining any new insights from the additional data.
4. Bias and variance trade-off: As more data are added, the model’s variance may decrease, but its bias may increase, resulting in a trade-off between the two that limits the improvement in accuracy [51].
5. Saturation of the underlying distribution: Similar to data redundancy, the model’s accuracy can plateau if the additional data do not introduce new patterns or relationships that the model has not already learned from the existing data. The model may have already learned all the useful information from the existing data, and further data points do not contribute much to its accuracy.

Figure 3 also shows that the training time for the random forest models increases in relation to the volume of data provided. In general, the training time of a random forest model can increase as more training data are provided, due to the increased complexity of the model and the larger size of the dataset. For example, as more training data are provided, the trees may need to grow deeper to capture the additional complexity of the data, which can increase the training time. Another reason is that random forests use bagging, which means that each tree is trained on a bootstrapped sample of the training data. As more training data are provided, the size of each bootstrapped sample increases, which can increase the training time. Training time increases when employing semi-supervised learning or active learning methods that apply iterative processes to retrain the model with new labelled data. However, none of the models’ training times exceeded 0.2 s when developed using supervised learning, indicating that the training of multiple random forest models should be feasible without becoming overly computationally demanding or time-consuming.

### 3.2. Semi-Supervised Learning Results

**Figure 5:**
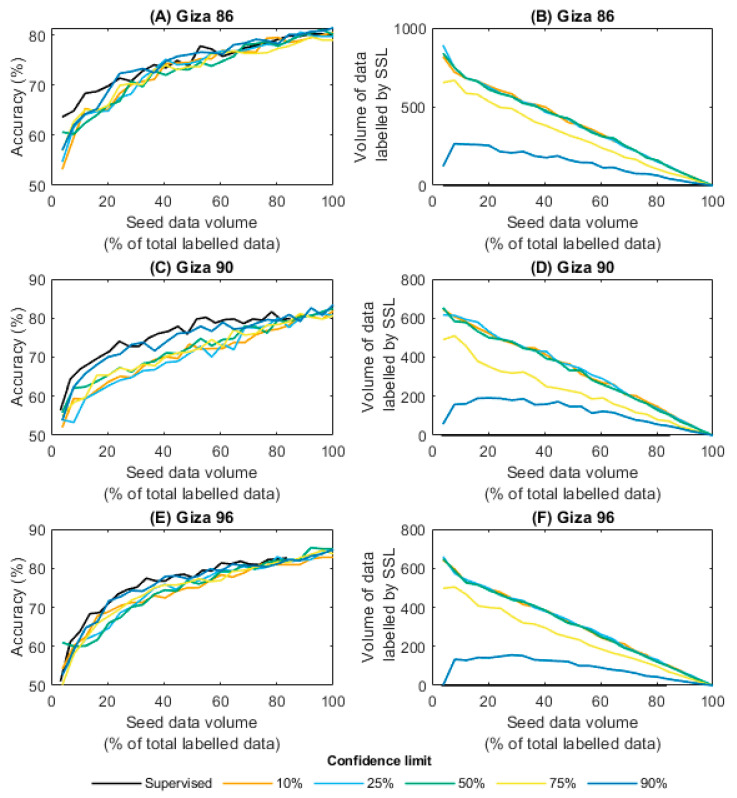
For varying training-image quantities (“seed data volume”) and cotton cultivars, the accuracy of predictions from the semi-supervised learning (SSL) model and the amount of data labelled through SSL are evaluated across five confidence thresholds.

Figure 5 depicts a comparison of the accuracy of classification and training-time outcomes of the random forest models generated utilising a semi-supervised learning method, with varying volumes of labelled “seed data” and confidence limits against the benchmark supervised-learning results. The outcomes represented in Figure 5A,C,E reveal that the utilisation of semi-supervised learning resulted in a marginal to non-existent rise in the classification accuracy for the Giza 86, 90, and 96 classification models. Furthermore, when compared with the supervised baseline model, the semi-supervised models of Giza 90 displayed a decrease in classification accuracy ranging from 3% to 11.5% until 400 seed data points, as shown in Figure 5C. This decrease was observed across confidence limits ranging from 10% to 75%.

There are several factors that may have caused the semi-supervised learning method to be ineffective. Firstly, adding more unlabelled data to a model can lead to overfitting if the model is too complex or the unlabelled data are noisy. Furthermore, semi-supervised learning relies on assumptions about the underlying data distribution, such as the smoothness assumption (i.e., points that are close to each other in the input space are likely to have the same output labels) or the cluster assumption (i.e., data contain clusters of points that correspond to different classes) [52]. As previously mentioned, Figure 4 reveals that the clustering of Egyptian cotton grades lacks clear definition, and it is possible for data points in proximity within the input space to correspond to distinct grades. This means that neither the smoothness assumption nor the cluster assumption holds true for the Egyptian cotton data, which may explain why the semi-supervised learning approach was not effective.

### 3.3. Active Learning Results

**Figure 6:**
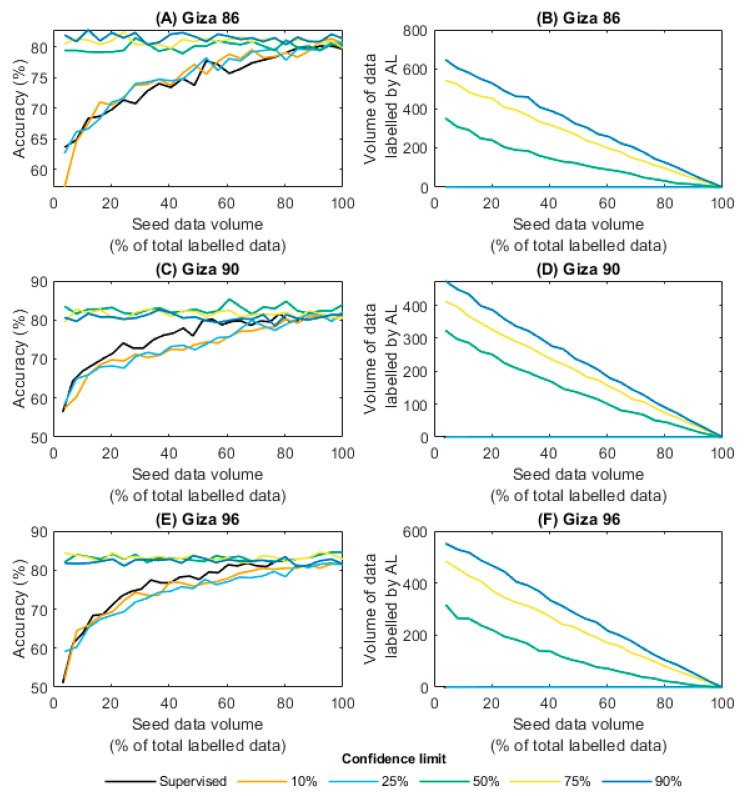
For varying quantities of training images (“seed data volume”) and cotton cultivars, the accuracy of predictions from the active learning (AL) model and the amount of data labelled through AL are evaluated across five confidence thresholds.

The results displayed in Figure 6 suggest that by selecting the most informative samples for labelling, active learning was able to achieve similar accuracy levels (78.50–85.33%) to the baseline supervised models (80.20–82.66%) trained on all the available data, but with significantly less labelling effort. Specifically, the most accurate active learning models reduced the volume of labelled data required by up to 46.4%, 38.9%, and 44.0% for the models built for Giza 86, 90, and 96, respectively. This is particularly beneficial for tasks that require expert annotation or labelling, such as the manual grading of cotton lint samples by CATGO human experts. Another key finding was that the best active learning models outperformed the best supervised models. For example, the active learning models with the highest accuracy for each cultivar (Giza 86: 82.85%, Giza 90: 85.33%, and Giza 96: 84.54%), were more accurate than the best results recorded for the supervised models (Giza 86: 80.20%, Giza 90: 81.62%, and Giza 96: 82.66%). This is likely to have been due to the fact that active learning selects the most informative samples for labelling, which improves the quality of the training data and leads to better model generalisation [42].

Active learning did result in increased training time, as the model needed to be retrained with each new sample added. The maximum training time for the supervised learning models was between 0.15–0.19 s, whereas the maximum training time for the active learning models was between 7.92–11.13 s. However, this is still very quick and would not hinder industry adoption. Furthermore, it was observed that starting with a smaller volume of seed data allowed active learning to effectively identify which samples required labelling, further reducing the overall volume of labelled data required. The time savings associated with having to label fewer samples would render the increase in model training time negligible. For example, when considering that either labelling the samples manually or by HVI requires approximately 30 s per sample, the total time taken to label the samples for each cultivar would be 422.5 min, 351.5 min, and 356.5 min for the Giza 86, 90, and 96 samples, respectively. The actively learning models were able to reduce the total volume of labelled data required by up to 58.0%, 51.2%, and 56.5% for the Giza 86, 90, and 96 models, respectively. Due to the negligible increase in model training time, the total time saved by using active learning was 245 min, 180 min, and 201.5 min for the Giza 86, 90, and 96 models, respectively.

The objective of active learning is to identify an effective strategy that can efficiently select the most informative samples, while minimising the required labelling effort. In this study, a strategy of uncertainty sampling was employed to identify samples for manual labelling, based on a pre-defined confidence limit that was determined by the model’s prediction confidence. The trade-off between the cost of acquiring additional labelled data and the expected improvement in model performance is determined by the confidence limit. This study investigated the impact of varying confidence limits on both model accuracy and the amount of labelled data required. As expected, increasing the confidence limit was associated with greater model accuracy, but this improvement came with an increase in the need for labelling and extended training duration. Interestingly, the confidence limits of 10% and 25% did not require any manual labelling, implying that active learning did not take place at these levels. When comparing the models built with confidence limits of 50%, 75%, and 90% and the lowest volume of total labelled data, all models achieved similar accuracy for each cultivar (Giza 86: 79.22–82.16%, Giza 90: 79.35–81.61%, and Giza 96: 81.83–83.95%), but the 50% models required fewer labelled data points (Giza 86: 355, Giza 90: 343, and Giza 96: 310) compared with the 75% (Giza 86: 564, Giza 90: 434, and Giza 96: 480) and 90% models (Giza 86: 647, Giza 90: 490, and Giza 96: 547).

Although the strategy of uncertainty sampling was effective in this study, other active learning methods also exist, such as query by committee, diversity sampling, and active learning with deep generative models [53], and these could be explored in future work. Future research could also investigate alternative sampling strategies that can identify the most informative samples while minimising the effort required for labelling.

## 4. Conclusions

Image processing and machine learning has emerged as a promising solution to automate inefficiencies in the assessment of food and industrial crop quality. The effectiveness of the image-classification solution in assessing crop samples is contingent on reducing data-labelling costs. This is because the models require retraining annually on newly labelled data that accurately reflect the changes in crop-quality standards, which can vary due to environmental and ginning factors, harvest conditions, and varieties. This study aimed to compare the effectiveness of supervised learning, semi-supervised learning, and active learning for developing models to automate the grading of Egyptian cotton lint samples. The findings generated are transferable and can be employed to address quality-assessment challenges encountered when grading a wide spectrum of food and industrial crops.

Overall, the findings suggest that active learning can be an effective approach for training machine learning models to classify Egyptian cotton lint grade based on image features. The active learning models were able to achieve high accuracy with a lower volume of labelled data compared with supervised and semi-supervised models. Additionally, active learning models were able to identify the most informative samples for labelling, reducing the overall labelling effort required to achieve high accuracy. By reducing the volume of labelled data required, the actively learning models reduced the total model development time by up to 58.0%, 51.2%, and 56.5% for the Giza 86, 90, and 96 models, respectively. By using active learning, the developed models are more adaptable to seasonal changes to cotton lint grading standards and can achieve high accuracy with less labelling effort. Future research investigating alternative sampling strategies for active learning is required to identify the most informative samples while minimising the effort required for labelling.

## Figures and Tables

**Table 1:** The Egyptian cotton grading system and breakdown of the number of samples of each grade for each cultivar.

Egyptian Cotton Grade	Giza 86	Giza 90	Giza 96
*I—*Fully good	115	0	103
*II*—Good to fully good	118	100	0
*III*—Good	113	131	109
*IV*—Fully good fair to good	119	116	118
*V*—Fully good fair	150	124	97
*VI*—Good fair to fully good fair	115	131	102
*VII*—Good fair	115	101	120
*VIII*—Fully fair to good fair	0	0	0
*IX*—Fully fair	0	0	64

## Data Availability

The data that support the findings of this study are available from the corresponding author, [OJF], upon reasonable request.

## Supplementary Materials

The following supporting information can be downloaded at: https://www.mdpi.com/article/10.3390/s23218671/s1, Figure S1. Flow chart of the three machine learning methods (A) supervised learning, (B) semi-supervised learning, and (C) active learning.
