# Supplementary material for: AI-Assisted Cotton Grading: Active and Semi-Supervised Learning to Reduce the Image-Labelling Burden
Source: Sensors (Basel). 2023 Oct 24;23(21):8671. doi: 10.3390/s23218671 (PMC10647751; doi:10.3390/s23218671)
Supplement: Supplementary file 1 [file sensors-23-08671-s001.zip › sensors-2653968-supplementary.pdf]

Supplementary Information

AI-Assisted Cotton Grading: Active and Semi-Supervised Learning to Reduce the Image-Labelling Burden

Oliver J Fisher[1,2*], Ahmed Rady[1,2], Aly AA El-Banna[3], Haitham H Emaish[4], Nicholas J Watson[6,1]

[1]Food, Water, Waste Research Group, Faculty of Engineering, University of Nottingham, Nottingham, NG7 2RD, UK; [2]School of Chemistry and Chemical Engineering, University of Surrey, Guildford, GU2 7XH, UK; [3]Teagasc Food Research Centre , Ashtown, Dublin, D15 DY05, Ireland; [4]Department of Plant Production, Faculty of Agriculture, Saba Basha, Alexandria University, Alexandria Governorate 5424041, Egypt; [5]Department of Soils and Agricultural Chemistry, Faculty of Agriculture, Saba Basha, Alexandria University, Alexandria Governorate 5424041, Egypt; [6]School of Food Science and Nutrition, University of Leeds, Leeds, LS2 9JT, UK
*Corresponding Author: o.fisher@surrey.ac.uk

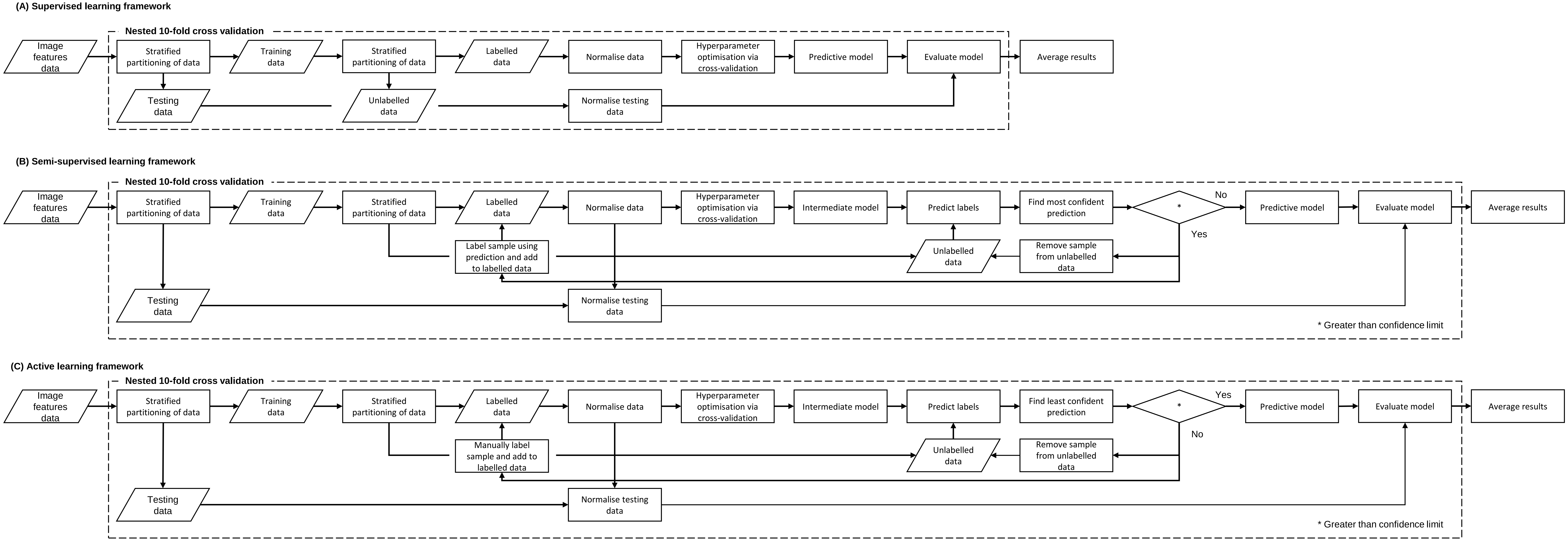

Figure S1. Flow chart of the three machine learning methods (A) supervised learning, (B) semi-supervised learning and (C) active learning
